# Analysis of Corrosion by Speckle Polarimetry

**DOI:** 10.3390/s25164941

**Published:** 2025-08-10

**Authors:** Francisco Gascón, Jorge Rodríguez, Ana Bayón, Francisco J. Nieves, Félix Salazar-Bloise

**Affiliations:** 1 Departamento de Física Aplicada II, IUACC, ETSA, ETS Arquitectura, Universidad de Sevilla, 41012 Sevilla, Spain; fgascon@us.es (F.G.); nieves@us.es (F.J.N.); 2 ETSI Minas y Energía, Universidad Politécnica de Madrid, Calle de Ríos Rosas 21, 28003 Madrid, Spain; j.rodriguez.ordonez@gmail.com (J.R.); anaisabel.bayon@upm.es (A.B.)

**Keywords:** speckle, polarization, acid corrosion, non-contact method

## Abstract

One of the most common problems in material engineering is the appearance of corrosion. For this reason, numerous efforts are underway to design materials that are resistant to this damage. In the same context, the diagnosis of corrosion is also of great interest since its detection reveals the real state of a structure. This article is focused on the latter. The purpose of the presented study is to provide a simple optical methodology to analyze the corrosion process and its evolution by means of a non-destructive method based on changes in the polarization state of the speckle patterns. To carry this out, two experimental arrangements with different wavelengths are proposed: one in the far field and another in the near field. The samples are first subjected to accelerated acid corrosion in the laboratory, and then, the degree of corrosion is quantitatively analyzed using the proposed technique. Moreover, in order to ensure that the acid attack on the samples is uniform (generalized corrosion), a detailed study is performed on the specimen surfaces via Raman spectroscopy. The results obtained show the ranges of applicability of both setups and their limits for studying corrosion.

## 1. Introduction

Corrosion can occur in several complex forms. Thus, the way in which a material is corroded depends on its specific physical and chemical characteristics, its shape, and the substances present in its surroundings. Some of the most common types of corrosion that can be distinguished include uniform or generalized, galvanic, pitting, crevice, graphitic, and microbiologically influenced corrosion. Each type exhibits unique characteristics. The causes of its appearance are very different, and the explanation of all phenomena involved is difficult to determine. Considering the importance of corrosion in so many industrial sectors, the need for its study is evident, as is the need for the development of measurement systems for its estimation and evolution. In this sense, there are numerous methods that allow corrosion to be analyzed.

One of the procedures for evaluating corrosion is the impedance method, which uses piezoelectric crystals [1,2]. Another evaluation procedure is the Electrochemical Noise Method (NM), which can be used in situ to investigate corrosion processes and to detect and monitor the corrosion of metallic materials [3]. Furthermore, among the methods for the study of corrosion, optical techniques are of particular relevance. These techniques are widely used due to their non-contact nature, high resolution, and ability to provide real-time monitoring.

Several optical approaches have been proposed to analyze corrosion. Optical microscopy (OM) has been used in its many different forms to examine surface morphology and detect corrosion pits, cracks, and uniform corrosion. High-resolution imaging provides qualitative analysis of corrosion effects, making it a valuable tool [4,5]. White Light Interferometry (WLI) is able to measure topography changes caused by corrosion at the nanometer scale, along with surface deterioration [6,7]. Raman spectroscopy (RS) is based on the inelastic scattering of light by phonons (vibrational spectroscopy) and is a highly accurate technique for identifying corrosion products. This method is also very sensitive to slight changes in surface properties and is particularly useful for characterizing oxide layers and determining the composition of rust and other corrosion-related compounds [8,9]. Laser Scanning Confocal Microscopy (LSCM) offers three-dimensional surface profiling with high depth resolution, thus being employed for measuring pit depth caused by corrosion, providing detailed information regarding the extent of surface damage [10]. Ellipsometry (EL) can detect changes in the polarization of reflected light in its interaction with a material. This characteristic makes it very useful for thin-film technologies, measuring the thickness of layers, and therefore, it is applicable to the study of corrosion product formation on metallic surfaces [11]. Fourier Transform Infrared Spectroscopy (FTIR) detects chemical changes on surfaces caused by corrosion. This technique is used to analyze protective coatings and corrosion inhibitors, helping in the selection and optimization of anti-corrosion treatments [12,13,14]. Holographic interferometry (HI) captures three-dimensional images of surfaces with high precision and allows for the analysis of the degree of corrosion and other related parameters by means of the interference fringes that appear in the experiment [15,16,17,18]. Focusing on optical methods, and with this article in mind, methodologies based on the speckle phenomenon are of great interest to us.

Speckle techniques have also long been applied for determining changes in the surface of metals, such as etch depth [19], variations in topography using via speckle field decorrelation [20], and fatigue detection in materials [21,22], as well as to analyze stress corrosion processes in the microregions [23]. Likewise, electronic speckle pattern interferometry (ESPI) is used to detect the existence of crevice corrosion, which is usually undetectable via visual inspection [24]. This technique reveals not only the location of defects but also their approximate sizes. Moreover, it is suitable for monitoring oxide layers growing in metallic structures [25] and for the detection of decohesion paints used in the industry [26].

The polarization properties of the speckle resulting from the interaction between the incident monochromatic radiation and the sample surface depend on the degree of corrosion of the material. In this context, this article aims to analyze corrosion and its evolution in a metallic sample (Fe steel) using speckle polarimetry. This study was conducted in the far and near fields, and the analysis using the degree of polarization (DOP) was complemented with information from point-by-point polarimetric histograms. In addition, this study shows the influence of wavelength on the experiments. More specifically, we show that the measurement of the changes in the polarization state of the scattered light is an efficient methodology for characterizing the corrosion process.

## 2. Materials and Methods

Monochromatic beam propagation through space is associated with a polarization state. This polarization may be understood as the result of the correlation between the two orthogonal components of the electric field. However, when light interacts with a rough material on the wavelength scale, due to the processes of single and/or multiple scattering, it can undergo variations in its polarization. This characteristic is fundamental, allowing us to carry out the objective of this paper.

**Figure 1:**
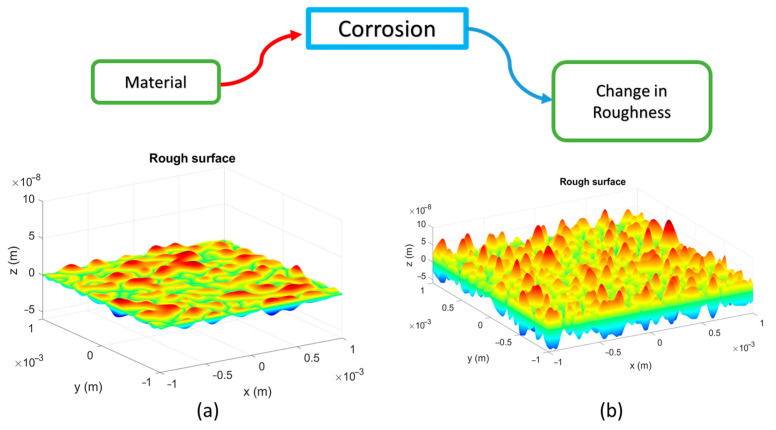
Material surface. (**a**) Rough surface of a sample. (**b**) Surface corroded. Corrosion produces changes in its topography (roughness).

**Figure 2:**
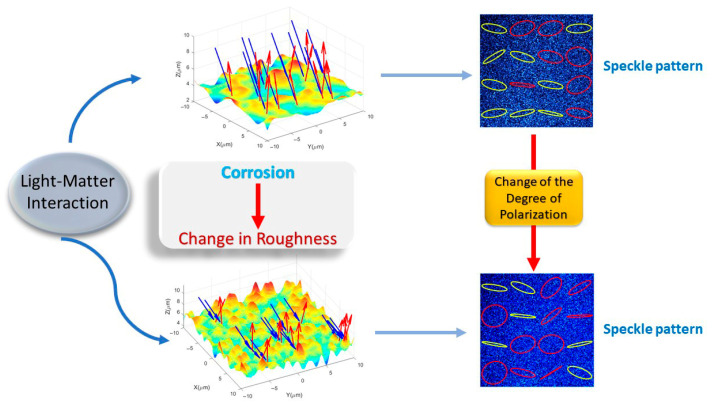
Diagram of the light–matter interaction. Corrosion modifies the surface structure, resulting in changes in the DOP of light.

One of the immediate mechanical effects of the appearance of corrosion on the material surface is the change in roughness (Figure 1). For this reason, if a laser beam is impinged on a corroded sample, the characteristics of the speckle produced should be different compared to the speckle pattern before corrosion occurred. In other words, corrosion changes the roughness, which in turn modifies the light–matter interaction process, resulting in a change in the polarization state of the spatial fluctuations of the field (speckle) (Figure 2).

### 2.1. Sample Preparation

The corrosive process of a material is a complex mechanism, whose evolution depends on numerous factors (components present in the environment, humidity, temperature, etc.). This process usually involves a lot of time in actual conditions. In order to study corrosion and its evolution at the laboratory scale in within a sensible amount of time, experiments were carried out to produce controlled generalized corrosion in an accelerated manner. The specimens employed were six slender prismatic metal bars (low-carbon steel) of 34.8 × 0.5 × 0.5 cm in size and with a surface area of 70.1 cm^2^ corroded via immersion in dilute nitric acid of different concentrations (see Appendix A). Before immersion, the samples were treated with hexanol. This substance is suitable for removing greasy residues and evaporates without leaving any traces. Furthermore, it does not attack metal.

At the same time, for this investigation, it was necessary to known in advance the degree of corrosion induced on the specimens; these reference data would allow us to evaluate the validity of the results obtained using the proposed optical method. To achieve this, a technique must first be chosen to quantify the degree of corrosion. As already mentioned in the Introduction, there are numerous methodologies that permit the qualitative/quantitative detection of corrosion. Thus, in this study, we applied the weight-loss method, which is an effective technique that can be used in any laboratory [27,28,29]. Following this technique, a 0.1 mg precision balance was used to measure the mass of each sample before and after corrosion caused by the acid, thus obtaining the mass loss per unit area.

### 2.2. Near and Far Fields

It is well known that when a rough surface is illuminated with monochromatic radiation, a random diffraction pattern, called speckle, is produced. The reason for this lies in the characteristics of the surface topography, i.e., the roughness, and the wavelength of the incident light. An important aspect in this light–matter interaction process is that when the light diffracted by the sample is observed far from the surface, its properties are not necessarily the same as those of the radiation near the specimen, since the light in its propagation can modify some of its initial properties. In fact, it has long been known that the spectral degree of coherence of the electric field, spectral degree of polarization, and spectral density (the spectrum) of the light may change under propagation, even in free space [30,31,32]. This is why an analysis of these two situations is important. However, in order to study this phenomenon, it is first necessary to define what is meant by near field and far field. Most authors consider near field to correspond to distances *d* from the surface that are smaller than the wavelength, whereas far field means distances o much greater than the wavelength (2.5–20λ). One of the main differences between these two regimes is that for *d* < λ, the electric field contains both propagating (homogeneous) and evanescent (inhomogeneous-short range) components, while for distances *d* > λ, only the homogeneous component appears.

In this paper, the viewpoint is a little different due to our experimental layout (depth of field >> λ). We will consider near field those distances very close to the surface (Fresnel number F >> 1), but where Fresnel’s quadratic approximation (paraxial) does not work, and we would need to use the Kichhoff-Fresnel integral. At the same time, this distance is far enough away from the specimen so that any analysis of evanescent waves is excluded. This scattered field will be analyzed by collecting it with an image system. In this context, far field may be understood as distances where the usual Fresnel and Fraunhofer approximations apply.

### 2.3. Experimental Setup and Modus Operandi

The basic idea of this study is to relate the changes in the polarization state of light when interacting with the material to the surface corrosion. This study analyzed the speckle produced by the sample in two different situations: one in the far field and the other in the near field. Thus, by analyzing the polarization in both cases, we will know which of the two configurations is the most adequate to obtain information on the corrosion of the material.

#### 2.3.1. Far Field Setup

**Figure 3:**
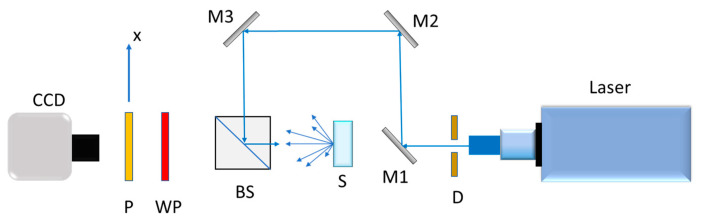
Setup to measure the DOP of the scattered light in the far field region.

Figure 3 shows the optical assembly used to determine the degree of polarization of the radiation scattered by the samples. The system consists of an HeNe laser of 632.8 nm with an expander; a diaphragm D to control the diameter of the incident beam on the sample; mirrors M1, M2, and M3 to redirect the beam; a beam splitter BS; a λ/4 waveplate (WP); a polarizer (*p*); and a CCD camera. The reason for this three-mirror structure is the operational versatility of the optical device to also work in transmission for experiments with translucent samples (this is not our case), achieved simply by eliminating mirror M1. This arrangement allows us to illuminate the sample perpendicularly and to collect the diffracted radiation data with a camera. The distance also between the material and the detector was chosen so that the speckle size recorded was larger than the CCD pixel extent.

Once the system was adjusted, the degree of polarization (DOP) was determined by measuring the Stokes parameters. As these parameters are directly related to the intensity of light, they can be measured by manipulating the information of the light scattered by the sample. Stokes parameters may be determined using the aforementioned elements: *p*, WP, and a camera (Figure 3). Thus, the parameters are found by measuring intensities in four steps, by rotating the polarizer and employing the waveplate (retarder).

**Figure 4:**
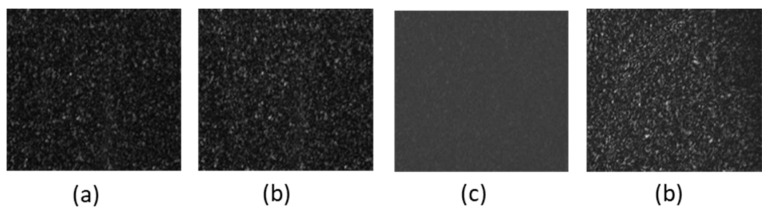
One example. Four pictures of the speckle intensities measured in one experiment with λ = 632 nm. (**a**) I(0°,0°). (**b**) I(45°,0°) . (**c**) I(90°,0°). (**d**) I(45°,90°).

Angle *θ* denotes the angle of the transmissive axis of the polarizer with respect to the chosen X-axis, and by placing the fast axis of the waveplate parallel to this axis, the required four different intensities to be measured can be denoted as *I*(0°,0°), *I*(45°,0°), *I*(90°,0°), and *I*(45°, 90°) (Figure 4). Once these intensities are known, the Stokes parameters, denoted by *S*_0_, *S*_1_, *S*_2_, and *S*_3_, can be expressed as a function of the intensities as follows [33]:

(1) $$\begin{array}{c} S_{0}=I\left(0{}^{\circ},0{}^{\circ}\right)+I\left(90{}^{\circ},0{}^{\circ}\right)\\ S_{1}=I\left(0{}^{\circ},0{}^{\circ}\right)-I\left(90{}^{\circ},0{}^{\circ}\right)\\ S_{2}=2I\left(45{}^{\circ},0{}^{\circ}\right)-I\left(0{}^{\circ},0{}^{\circ}\right)-I\left(90{}^{\circ},0{}^{\circ}\right)\\ S_{3}=2I\left(45{}^{\circ},90{}^{\circ}\right)-I\left(0{}^{\circ},0{}^{\circ}\right)-I\left(90{}^{\circ},0{}^{\circ}\right) \end{array}$$

Parameter *S*_0_ represents the total intensity of the light. *S*_1_ describes the amount of linear or vertical polarization, and *S*_2_ is the amount of linear 45° polarization or 135° linearly polarized light. *S*_3_ gives the amount of right- and left-handed circularly polarized light of the beam. From these quantities (1), the degree of polarization is defined as

(2) $$P=\frac{\sqrt{S_{1}^{2}+S_{2}^{2}+S_{3}^{2}}}{S_{0}}$$

where 0≤P≤1. This means that if *p* = 0, the light is unpolarized, and when *p* = 1, the radiation is completely polarized; for any other intermediate value, the light is partially polarized.

#### 2.3.2. Near Field Setup

**Figure 5:**
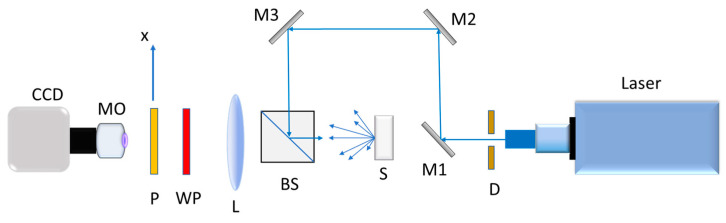
Layout to measure the speckle pattern in the near field.

The experimental device used to determine the degree of polarization (DOP) of the near-field speckle is shown in Figure 5.

The basic structure is similar to that shown in the previous section. However, in this optical assembly a lens microscope objective system was incorporated to form an image of the sample, and an Argon laser was used. In this way, the CCD records the speckle produced in the vicinity of the surface of the material, allowing us to measure the degree of polarization in the near field. The optical layout consists of a lens L and a microscope objective MO. This allowed us to enlarge the speckle diameter as much as needed for the experiment, avoiding pixel size problems. This is important for carrying out the calculations well. Once the optical system is adjusted, the procedure used to calculate the degree of polarization of the speckle produced by the rough sample is the same as that described in the previous subsection. Then, the intensity detected at four different positions of the polarizer and the analyzer is measured.

## 3. Results

### 3.1. Corrosion of the Samples

As mentioned in Section 2.1, samples were prepared in advance in order to perform the experiments. To induce controlled corrosion, the experiments were carried out with different acid concentrations and for different durations. The specimens were immersed in their respective cuvettes in dilute nitric acid solutions of 0.1 M, 0.5 M, 1 M and 2 M, at 20 °C, for 10, 20, 30, and 40 min, respectively. The selection of an acidic environment allows for the acceleration of the surface degradation process and for research material to be obtained quickly using the method proposed.

However, it also creates certain problems, especially in the case of nitric acid, related to the change in the digestion mechanism with increasing acid concentration. Another problem associated with the use of acid as an aggressive environment is the activation of the sample surface to secondary reactions that may occur on the surface.

The samples were cleaned with hexanol before immersion. After that, the specimens were washed with water and then dried in an oven to remove any remaining residues. In this manner, the knowledge of the acid’s effect on the samples allows us to better choose the series of specimens with which to carry out the optical experiments.

**Figure 6:**
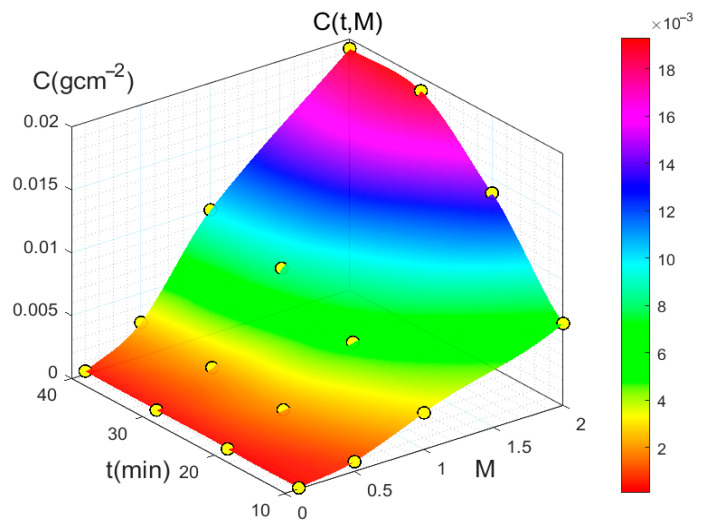
Mass loss per unit area associated with corrosion C as a function of time t that the sample was exposed to acid and its molarity M.

The results obtained for the samples subjected to corrosion are shown in Figure 6 (see Section 2.1). The first graph presents the values of the mass loss per unit area associated with corrosion, as a function of the time that the sample remains exposed to the acid for different molarities (C = C (t, M)). As expected, it shows an increase in the degree of corrosion as the molarity and the time increase.

Once the effect of the acid on the metal samples was known, five specific samples (called MI, I = 0, 1, … 5) were prepared using the 1 M solution. We chose the 1 M concentration because it caused corrosion levels in a wide interval, allowing us to study the validity range of the optical method well. The experiments were carried out with these samples carefully and were not performed in triplicate, as usual. However, this does not invalidate the analysis, since the weight losses reported exceed the mass that would have resulted due to sampling error.

**Figure 7:**
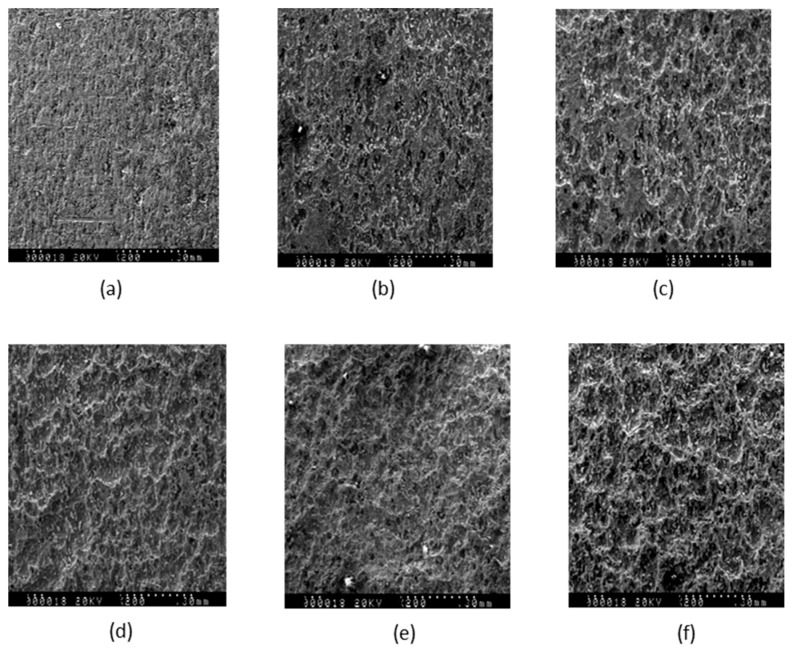
SEM images corresponding to the samples used in the experiments (20 kV; magnification: 200; area: 300 × 350 μm^2^). The values show the mass loss per unit surface (in mgcm^−2^) and the exposure time to the acid solution (in minutes), respectively. (**a**) Sample M0, not corroded. (**b**) M1 (13.6, 6.4). (**c**) M2 (22.8, 11.4). (**d**) M3 (33.3, 16.6). (**e**) M4 (51.2, 27.1). (**f**) M5 (68.4, 39.1).

In order to corroborate that the accelerated corrosion was of a generalized type, a surface study was carried out using SEM microscopy. The results obtained for the samples above are shown in Figure 7: Figure 7a shows the initial sample without corrosion. In Figure 7b, a change in the surface due to exposure to acid can be observed. In Figure 7c, a notable change in the surface structure may be seen. Finally, in Figure 7d–f, an increase in roughness can be observed between them, although not as pronounced as in the first three.

As can be observed, the change in the surface structure is quite homogeneous and, in addition, an increase in the erosion caused by the acid is detected as its concentration increases. In order to be sure that after the contact of the specimens with the diluted acid the properties of their surfaces remain uniform in composition, free of dirt or additional elements, a study using Raman spectroscopy was carried out.

Raman spectra of the specimens were recorded in the range from 80 to 3500 cm^−1^ using an alpha300 RS-Raman-SNOM Microscope (WITEC GmbH, Ulm, Germany), equipped with a 532 nm excitation laser at 35 mW laser power.

**Figure 8:**
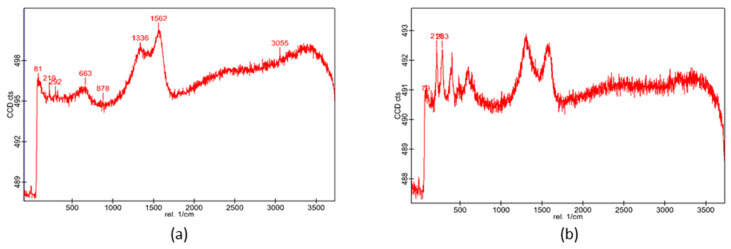

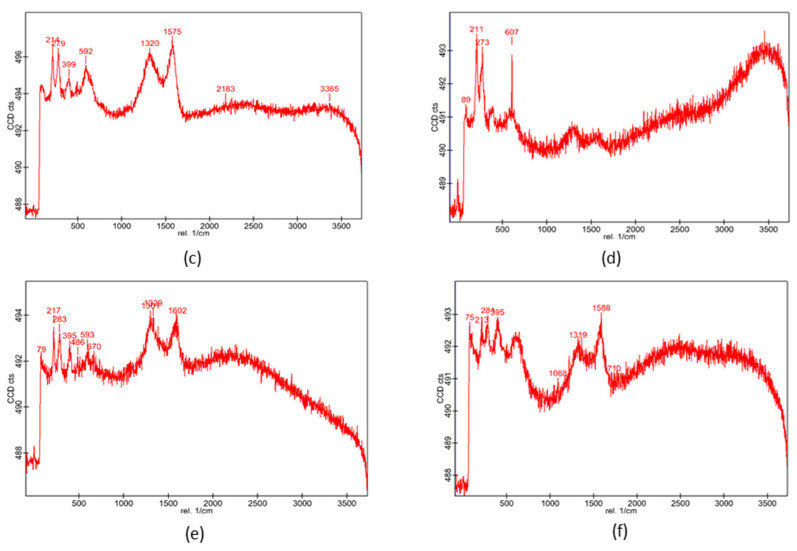
Raman analysis of the samples shown in Figure 4. The values depict the mass loss per unit surface (mgcm^−2^) and the exposure time to the acid solution (minutes), respectively. (**a**) Specimen M0, without induced corrosion. (**b**) M1 (13.6, 6.4). (**c**) M2 (22.8, 11.4). (**d**) M3 (33.3, 16.6). (**e**) M4 (51.2, 27.1). (**f**) M5 (68.4, 39.1).

The study was carried out by analyzing points at intervals of 20/1024 throughout 1024 lines over areas of 20 μm^2^, in different zones for each sample, using filters to treat the signal and to favor the extraction of the intense peaks. All results are summarized in Figure 8.

The first picture (a) shows the Raman shift in the initial sample without corrosion. As can be seen, there are various peaks in the spectrum (219, 292, 663, 1336, and 1562 cm^−1^) that are compatible with the existence of Fe_2_O_3_. This could indicate that, although this initial sample was not exposed to the artificial corrosion process in the laboratory, the steel itself already had oxidized zones, which can be due to multiple causes related to the interaction of the material with the external environment, including its manufacturing process. This reveals that the appearance of rust is common in this type of material, which is easily attacked by substances present in the environment (dust, contaminants, humidity, etc.). Figure 8b shows the results for the first sample attacked by the solution (see Figure 7b). Figure 8c corresponds to specimen M2, for which almost the same intensity peaks are observed, although one new peak appears around 400 cm^−1^. This value is not usually associated with individual elements per se, but rather to compounds or phases that contain certain elements. For this reason, it is difficult to be sure exactly to which substance it refers. There are different possibilities; however, in our case, as the samples comprise steel, the peak probably corresponds to iron oxides and hydroxides, since these components occupy a band of the spectrum that contains this value. In Figure 8d the spectrum is again similar to the previous ones, with more noise overlapping some small peaks. Finally, in Figure 8e,f, the same general behavior as in the previous figures is observed. More numerical values appear on the graphs, which are the data processing results; these are irrelevant to the present study. It should also be mentioned that in all the pictures, a wide continuous spectrum appears from 1700 cm^−1^. This is a very wide range in which mainly molecular or surface-bound groups are detected, but not elemental vibrations. These molecular features indicate the presence or involvement of certain elements or functional groups. In particular, the final part between 3200 and 3600 cm^−1^ could show the presence of water, hydroxyls, or carboxylic acids.

### 3.2. Optical Measurements

In order to verify the validity of this research, numerous far field and near field experiments were carried out using the optical systems described above. In addition, different wavelengths were used to investigate their influence on the results. For this purpose, six samples with different known degrees of corrosion were prepared.

#### 3.2.1. Far Field Measurements

**Figure 9:**
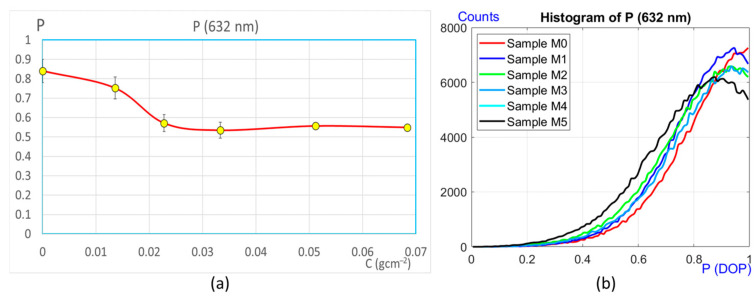
Degree of polarization (DOP). (**a**) Functional dependency of *p* against corrosion C. (**b**) Histograms corresponding to the DOP (point by point) for each sample.

These experiments were carried out using the device shown in Figure 3, with a HeNe laser of wavelength 632 nm. The results obtained are presented in Figure 9.

The graph in Figure 9a shows the degree of polarization (DOP) as a function of corrosion for each of the samples. These results clearly show how the polarization of the scattered light varies as the corrosion increases. It is observed that in the first stages of the corrosion process analyzed (samples M0, M1, M2, and M3), the polarization changes in a very appreciable way. However, thereafter, the degree of polarization undergoes small variations, and the relationship between the two quantities *p* and C cannot be clearly correlated. It follows that this device (far field), with this wavelength, allows us to study the corrosive process of the samples in the initial stages (Figure 7a–d). Subsequently, the DOP fluctuates around the same value, making it difficult to correlate it well with corrosion, i.e., relative variations in the DOP cannot be reliably distinguished. However, another DOP study can be performed parallel to the previous one, which could provide information not obtained with the previous analysis. To do so, it must be considered that the calculation of the DOP performed is a global analysis of the polarization. In other words, what has been done is the average DOP measurement of the speckle pattern contained in the whole detector. The idea now is different. It involves obtaining the DOP at each point of the image (pixel), calculating its histogram, and relating it to the degree of corrosion. In other words, the DOP is calculated point by point..

**Figure 10:**
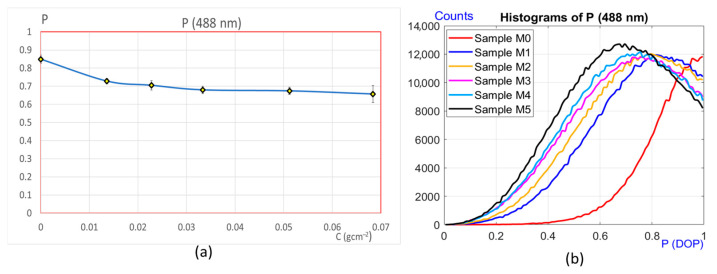
Experimental results with λ = 488 nm. (**a**) Degree of polarization (DOP) versus corrosion. (**b**) Histograms corresponding to the DOP, point by point.

In performing this calculation with the same speckle patterns, their corresponding histograms can be seen in Figure 9b. Differences between the histograms are observed for each of the samples, but these variations do not seem to follow any clear sequence (except for M0 and M1, at the beginning). Therefore, no extra information can be extracted regarding the degree of corrosion. From all this, we can observe that this second option of the DOP analysis (in this case) does not provide more information than that obtained with the global calculation of the polarization. However, it is worth mentioning that the range corresponding to the first four samples is actually a strong corrosion range (0–0.03 gcm^−2^ of mass loss per unit area). In a real application where the corrosion process is slow, and where the corrosion is often weak, the proposed technique would be useful to study the corrosion changes, since corrosion values higher than those of sample M3 are not reached in many cases. Only in the case of aggressive corrosion caused by highly acidic environments would the proposed method attract less attention, since it would only provide information at the beginning of the process (Figure 10a), as it is unable to distinguish the evolution for high corrosion (samples M4 and M5).

#### 3.2.2. Measurements in the near Field

In a similar way as explained previously, the experiments were conducted with the same samples, but using the arrangement shown in Figure 5. Unlike in the previous case, the measurements were performed with wavelengths of 488 nm and 514 nm, corresponding to an argon laser. These wavelengths have good power and coherence length. The results obtained are presented in Figure 10. Figure 10a depicts the behavior of the DOP against corrosion for λ = 488 nm. A decreasing function is obtained for all the corrosion values studied, and it can be distinguished how the polarization state changes for all the samples. Only specimen M3 is similar to M4. In this case, there is ambiguity between their values, because they exhibit almost the same corrosion. Proceeding in the same manner as before, DOP histograms were obtained for each point (pixel), as shown in Figure 10b. As may be observed, the set of graphics follow a clear sequence. The histograms shift to the left as corrosion increases. It is of interest to note that the largest change is observed between sample M0 (non-corroded) and M1 (corroded).

This indicates that the proposed method is very sensitive in detecting the beginning of the corrosion process. In addition, the method is also able to eliminate the ambiguity that existed between samples M3 and M4 (Figure 10a). Indeed, the histograms corresponding to these samples are now well identifiable (in magenta (M3) and in light blue (M4)). Therefore, we can say that the measurement of the global DOP together with its statistical distribution per pixel represents a good methodology for the investigation of corrosion in the interval of interest. The degrees of corrosion of each of the samples are identifiable and distinguishable; therefore, the near field measurements employing this wavelength allow us to study the evolution of the corrosion of the material over a wider range than in the near field.

**Figure 11:**
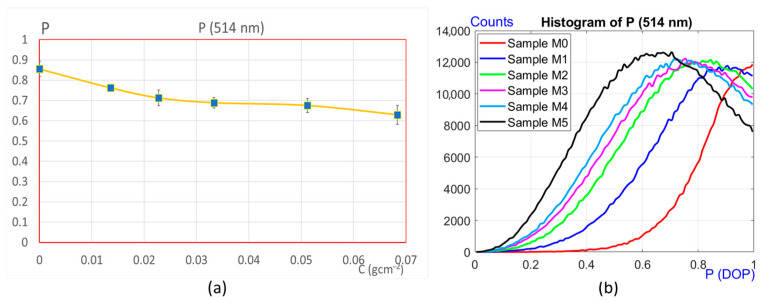
Experimental results with λ = 514 nm. (**a**) Degree of polarization against corrosion. (**b**) DOP histograms.

With the optical layout described above (Figure 5), measurements were taken using a monochromatic radiation of λ = 514 nm, and the results are shown in Figure 11. The behavior of the degree of polarization *p* is again similar in structure to the previous one, but with other values and without ambiguity in any of them, i.e., each global DOP corresponds to a single point of corrosion in the graphic. Even though the differences in the DOP are appreciable over the entire range studied, *p* was also analyzed for each image point, the result of which is depicted in Figure 11b. The histograms obtained are remarkably different from each other. Note that they also follow a sequence clearly correlated with the corrosion. In the same way as for 488 nm, this is important because this 514 nm radiation also allows us to study corrosion without ambiguity over the target range.

From these results, it can be inferred that the analysis of the DOP of the speckle in the near field is more suitable than the methodology employed in the far field, since it allows the analysis of the corrosion of the material and its evolution over a wider interval.

## 4. Discussion

Measurements were carried out using two different optical devices. The first one allows the analysis of the degree of polarization (DOP) of the speckle in the far field, and the second one in the near field. The study was carried out on samples with different degrees of corrosion prepared in the laboratory. Comparing the graphs in Figure 9 with those in Figure 10 and Figure 11, it can be seen that the range of applicability of both techniques is different. In the first case with far field detection, with λ = 632 nm, the method proved to be suitable for detecting corrosion in its early stages, but as the corrosion increases, it reaches a point where it is no longer possible to quantify its evolution (samples M4 and M5). On the other hand, using the near field methodology, measuring the global DOP and with the histogram information, a wider range is available for the detection of corrosion.

**Figure 12:**
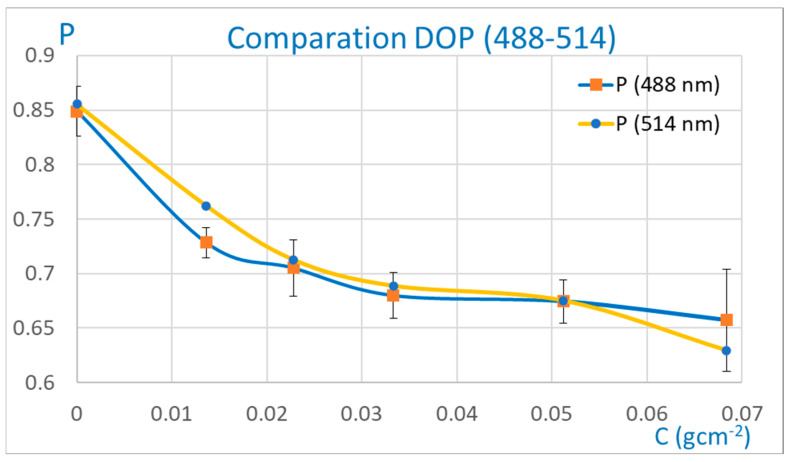
Experimental results (near field) with λ = 514 nm and λ = 488 nm.

In particular, focusing only on the results of the second layout (Figure 12), it can be seen that measurements taken with λ = 514 nm provide more information about the corrosion status of the samples than those corresponding to λ = 488 nm. It should be noted that with λ = 514 nm, it is possible to quantify the degree of corrosion unambiguously over a wider range (0–0.068 gcm^−2^), with the global DOP alone, and without the need of histograms.

It is interesting to comment on the reason why the information obtained using the two optical experimental arrangements is different. In the analysis in free space geometry (speckle pattern in the far field, first setup), the speckle detected with the CCD array is due to the random interference of the light scattered by the whole surface. In other words, the intensity recorded at an arbitrary point of the detector (pixel) is due to the interference of each of the waves coming from each of the points of the illuminated surface, which in our case was 4.5 mm in diameter, much larger than the camera’s pixel size. In the case of image geometry (near field, second arrangement), the detection corresponds to the speckle intensity close to the surface, at each point. This means that although the sample is illuminated over its entire surface, the reflected light falling onto a point near the surface, forming its image on the sensor (detector), will come only from a small area (very localized) of the specimen. This area is determined for the PSF of the optical system, and in the case examined, it corresponds to a pixel in the image plane, approximately. This shows that the near field intensity provides information about what is happening at each point of the surface and its surroundings. For this reason, it seems to logical that the results of the DOP for both arrangements are different, even though they have a similar trend in the early stages of the corrosive process (samples M0–M3).

## 5. Conclusions

In this work, two different optical methodologies were shown that allow us to determine the degree of corrosion and its progression (evolution). Overall, both techniques are valid for this objective, although they differ in their application ranges. Both methods are based on the measurement of the degree of polarization of the speckle pattern generated through the interaction of monochromatic radiation with the rough surface of the material. In addition, different types of radiation were used to determine the possible influence of the wavelength on the results. It was shown that this DOP analysis can be carried out in two complementary ways using the same images without more experiments. In the first approach, the global (mean) degree of polarization of the speckle pattern is calculated. In the second, the DOP is computed at each detector point (pixel), and the histogram is determined. This parallel analysis of the degree of polarization may be important, since as a result of the analysis, multivalued experimental data can be distinguished. Taking all this into account, as well as the results obtained, we can conclude that the near field analysis (image geometry) seems to be more suitable for the proposed objective than the far field study (free space geometry), in the interval chosen (0–0.068 gcm^−2^). Regarding the influence of the wavelength in the near field, λ = 514 nm seems to be better than λ = 488 nm, since all corrosion values obtained with the former correspond to well-differentiated degrees of polarization DOPs. However, considering that in all cases, both complementary procedures of calculating the DOP are used (global and point by point), it can be conjectured that both wavelengths are equally valid for the near field study of the corrosion process.

The methodology is equally applicable to curved surfaces, as it is based on changes in the degree of polarization. The main limitation of the system is its stability. Regarding the repeatability of the samples’ results, experiments must be carried out under the same conditions, guaranteeing, in principle, the results’ reliability at the laboratory scale.

## Data Availability

The data are contained within the article.

## Abbreviations

The following abbreviations are used in this manuscript:

DOP	Degree of polarization
M0	Sample 0
M1	Sample 1
M2	Sample 2
M3	Sample 3
M4	Sample 4
M5	Sample 5

## Appendix A

Nitric acid is a strong oxidizing acid that can cause corrosion in many metals, especially at higher concentrations and temperatures. In order to control the degree of corrosion as well as its homogeneity, dilute solutions of nitric acid were used. This type of corrosion is known as hydrogen corrosion. It typically occurs in acidic environments such as acidic industrial water and in solutions of monooxidizing acids. The anodic reaction involves iron ions dissolving according to the following reaction:

$$Fe\to Fe^{2+}+2e^{-}$$

The cathodic reaction consists of the elimination of hydrogen according to the following half-reaction:

$$2H^{+}+2e^{-}\to H_{2}$$

The overall reaction, therefore, is written as

$$Fe+2H^{+}\to Fe^{+2}+H_{2}↑$$

For a solution with nitric acid, this is not the last state of the oxidation of iron, as it still undergoes the following reaction:

$$3Fe^{+2}+NO_{3}^{-}+4H^{+}\to Fe^{+3}+H_{2}O+NO$$
